# Transcatheter radiofrequency pulmonary artery denervation in swine: the evaluation of lesion degree, hemodynamics and pulmonary hypertension inducibility

**DOI:** 10.1186/s12890-021-01786-y

**Published:** 2021-12-18

**Authors:** Natalia S. Goncharova, Heber Ivan Condori Leandro, Aleksandr D. Vakhrushev, Elena G. Koshevaya, Yury A. Skorik, Lubov B. Mitrofanova, Lada A. Murashova, Lev E. Korobchenko, Elizaveta M. Andreeva, Dmitry S. Lebedev, Olga M. Moiseeva, Evgeny N. Mikhaylov

**Affiliations:** Almazov National Medical Research Centre, 2, Akkuratova Str., Saint-Petersburg, Russian Federation 197341

**Keywords:** Pulmonary hypertension, Pulmonary denervation, Radiofrequency ablation, Large animal models, Synthetic analogue of thromboxane A_2_

## Abstract

**Background:**

Mechanisms of positive effects of pulmonary artery (PA) denervation (PADN) remain poorly understood. The study aimed to evaluate pulmonary hemodynamic changes after PADN and their association with the extent of PA wall damage in an acute thromboxane A2 (TXA2)-induced pulmonary hypertension (PH) model in swine.

**Methods:**

In this experimental sham-controlled study, 17 normotensive male white Landrace pigs (the mean weight 36.2 ± 4.5 kg) were included and randomly assigned to group I (n = 9)—PH modeling before and after PADN, group II (n = 4)—PADN only, or group III (n = 4)—PH modeling before and after a sham procedure. Radiofrequency (RF) PADN was performed in the PA trunk and at the proximal parts of the right and left PAs. PA wall lesions were characterized at the autopsy study using histological and the immunohistochemical examination.

**Results:**

In groups I and II, no statistically significant changes in the mean pulmonary arterial pressure nor systemic blood pressure were found after PADN (−0.8 ± 3.4 vs 4.3 ± 8.6 mmHg, *P* = 0.47; and 6.0 ± 15.9 vs -8.3 ± 7.5 mmHg, *P* = 0.1; correspondingly). There was a trend towards a lower diastolic pulmonary arterial pressure after PADN in group I when compared with group III during repeat PH induction (34.4 ± 2.9 vs 38.0 ± 0.8; *P* = 0.06). Despite the presence of severe PA wall damage at the RF application sites, S100 expression was preserved in the majority of PA specimens. The presence of high-grade PA lesions was associated with HR acceleration after PADN (ρ = 0.68, *p* = 0.03). No significant correlation was found between the grade of PA lesion severity and PA pressure after PADN with or without PH induction.

**Conclusions:**

Extended PADN does not affect PH induction using TXA2. Significant PA adventitia damage is associated with HR acceleration after PADN. Possible delayed effects of PADN on perivascular nerves and pulmonary hemodynamics require further research in chronic experiments.

**Supplementary Information:**

The online version contains supplementary material available at 10.1186/s12890-021-01786-y.

## Background

The destruction of the neural elements in the pulmonary artery (PA) trunk and proximal parts of the right and left PAs is thought to abolish or significantly decrease the efferent sympathetic nervous supply of pulmonary vasculature and causes PA vasodilatation [1, 2]. Several clinical studies have demonstrated a substantial improvement in pulmonary hemodynamics and exercise capacity after pulmonary artery denervation (PADN) in patients with different etiologies of pulmonary hypertension (PH) [3–8], identifying interventional or surgical PADN as an attractive additional PH treatment option [9–13].

Large animals are used for the elaboration of the PADN technique and PA nerve lesion characteristics. However, hemodynamic effects should be interpreted with caution since acute models do not resemble chronic pulmonary arteries remodeling, and chronic models have different pathophysiology compared to humans [14]. The synthetic thromboxane A2 analogue-induced PH model suggests acute and dose-dependent PA pressure (PAP) elevation with complete PAP normalization after thromboxane A2 (TXA2) withdrawal [15, 16]. Acute stable controlled but reversible TXA2-induced PH could be a relevant type of PH modeling for acute PADN effects assessment.

Another limitation of in vivo studies is the lack of data on the association between the denervated PA surface area and the hemodynamic effects. Previous studies have reported on the limited possibility of transcatheter approaches for achieving deep PA lesions that result in perivascular nerve damage [17]. However, a recent study reported on acute radiofrequency (RF) PADN effects in swine with PH induced with a synthetic TXA2 analogue [18]. Rothman and co-workers have demonstrated that transcutaneous RF PADN in swine led to nerve fibers’ damage with the absence of S100 expression at the sites of RF ablation, accompanied by a mean pulmonary artery pressure (mPAP) decrease in TXA2-induced PH modeling. However, the number of RF applications was small (from 2 to 7 lesions per animal) and did not lead to circumferential PA wall damage, while the authors demonstrated a correlation between the number of RF ablation and the mPAP decrease.

It is still unclear what radiofrequency (RF) ablation parameters might result in an effective PAP decrease, the quantity and timing of the PA neural damage for the treatment goal need further evaluation. We have hypothesized that the ablated PA wall area during PADN might be important for the resulting mPAP decrease in the TXA2-induced PH model.

The study aimed at the evaluation of pulmonary hemodynamic changes after PADN and their association with the extent of PA wall damage in an acute TXA2-induced PH model in swine.

## Methods

### Experimental animals and study design

The experimental study comprised 17 normotensive male white Landrace pigs. All procedures and protocols were reviewed and approved by the Institutional Animal Care and Use Committee and performed in the Preclinical and Translational Research Center of the Almazov National Medical Research Centre. The animals were obtained commercially from the CJSC "Livestock farm Ruchyi" (Saint-Petersburg, Russia) and were allowed enough time to habituate prior to procedures. The study was reported in accordance with ARRIVE guidelines https://arriveguidelines.org.

According to the study aim, we evaluated possible pulmonary hemodynamic changes in three different animal groups: induced PH before and after PADN (group I, PH-PADN), before and after PADN without PH induction (group II, nonPH-PADN), repeated PH induction with sham PADN (group III, PH-shamPADN). All animals were randomly assigned in one of three groups in the 2:1:1 ratio: group I, n = 9; group II, n = 4; and group III, n = 4. The size of the group I animals was chosen based on previous research [16]. We hypothesized that PH induction together with endothelial lesion during RF ablation may result in complications and hemodynamic deterioration in a part of study subjects. On the other hand, the requirement on minimizing the total number of large animals for experimental procedures forced us to limit two control groups—group II and III. Thus, the size of the group I would be justifiable larger than other groups. Blocked randomization was performed using an online randomization service (https://www.sealedenvelope.com/simple-randomiser/v1/lists). Randomization allocation was revealed to researchers at the moment the procedure started. However, further autopsy study was performed by pathologists (EGK and LBM) blinded to allocation groups.

**Fig. 1:**
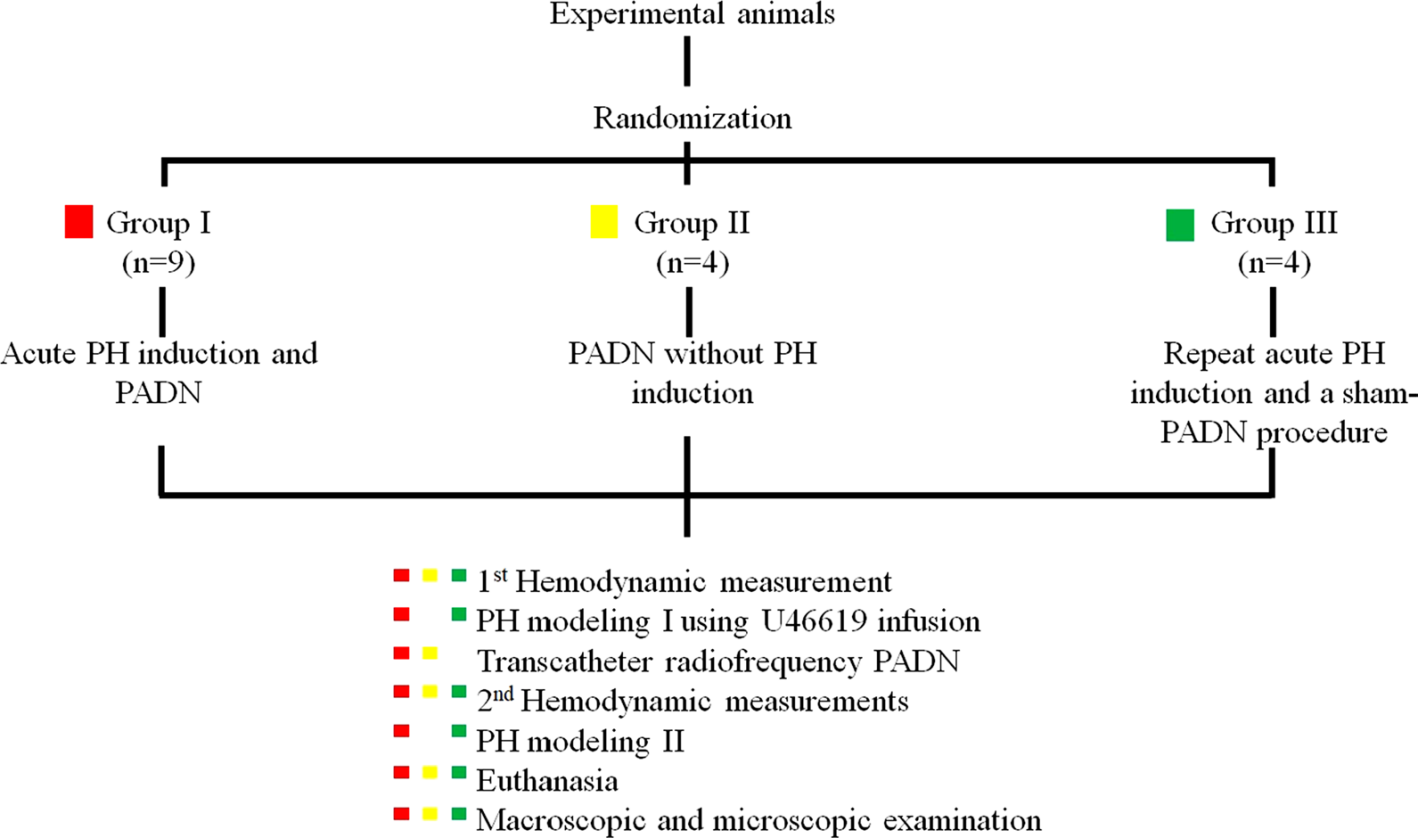
Study flowchart. RHC -right heart catheterization; PH-modeling-1—pulmonary hypertension induction using continuous TXA2 infusion before PADN; PH-modeling-2—pulmonary hypertension induction using continuous TXA2 infusion after PADN; PADN—pulmonary artery denervation; mPAP—mean pulmonary artery pressure

In group I (PH-PADN), the protocol consisted of the following phases: (1) baseline hemodynamic assessment with right heart catheterization (RHC), (2) reversible PH modeling-1 using TXA2 infusion, (3) transcatheter radiofrequency PADN, (4) hemodynamic measurements with RHC, (5) reversible PH modeling-2, (6) euthanasia, (7) pathology and immunohistochemistry studies of the PA (Fig. 1).

In group II (nonPH-PADN), the protocol consisted of (1) baseline hemodynamic measurements with RHC, (2) PADN, (3) repeat hemodynamic assessment with RHC, (4) euthanasia, (5) pathology and immunohistochemistry studies of the PA.

The following phases were considered in group III (PH-shamPADN): (1) baseline hemodynamic measurements with RHC, (2) reversible PH modeling-1, (3) a 60 min waiting period after hemodynamic normalization with catheter manipulations inside the PA and its branches, and RHC, (4) reversible PH modeling-2, (5) euthanasia, (6) autopsy study with PA pathology analyses.

### Experimental procedures

#### Catheterization and hemodynamic evaluation

The procedures were performed in a supine position under general anesthesia (tiletamine and zolazepam, 20 mg/kg, xylazine 3 mg/kg and atropine 0.1 mg/kg for induction, and isoflurane, 2 to 3%, with O_2_ via an endotracheal tube at 65%), and vascular access was obtained via the right and left jugular veins and right external carotid artery. A 7F vascular sheath (AVANTI® + , Cordis, Florida, USA) was placed into the right internal jugular vein. PA angiography was performed using a multipurpose angiographic catheter (Multipurpose, Cordis, USA) using a fluoroscopic C-arm (BV Endura C-Arm, Philips, Veenpluis, The Netherlands). RHC with the assessment of systolic pulmonary artery pressure (PAP), diastolic PAP, mPAP, mean right atrial pressure (mRAP), and pulmonary capillary wedge pressure (PCWP) was performed using the Swan Ganz catheter (Corodyn™P2, BRAUN, Bethlehem, Germany). Systemic BP was monitored and arterial blood samples were taken from the right external carotid artery. Arterial and PA blood gas analyses were performed using the I-STAT analyzer (Abbot, USA). Cardiac output (CO) was determined using the Fick method; pulmonary vascular resistance (PVR) and systemic vascular resistance (SVR) were calculated per the established equation formula (PVR = [80 × (mPAP—PCWP)/CO]). Hemodynamic measurements and monitoring were initiated after vascular sheaths placements and were continued throughout the procedure. Discrete measurements at baseline, at target mPAP during PH modeling-1, 20 min after PADN, and target mPAP during PH modeling-2 were performed. To prevent thrombotic complications, heparin sulfate was administered intravenously at a rate of 300 U/kg/h with the target ACT > 300 s. To maintain intravascular volume, an isotonic NaCl solution and succinylated gelatin (Gelosfusine®, Braun, Germany) were administered continuously; body temperature was maintained at 38 °C using a warming system (WarmTouch™, Medtronic, MN, USA).

#### Pulmonary artery denervation procedure

**Fig. 2:**
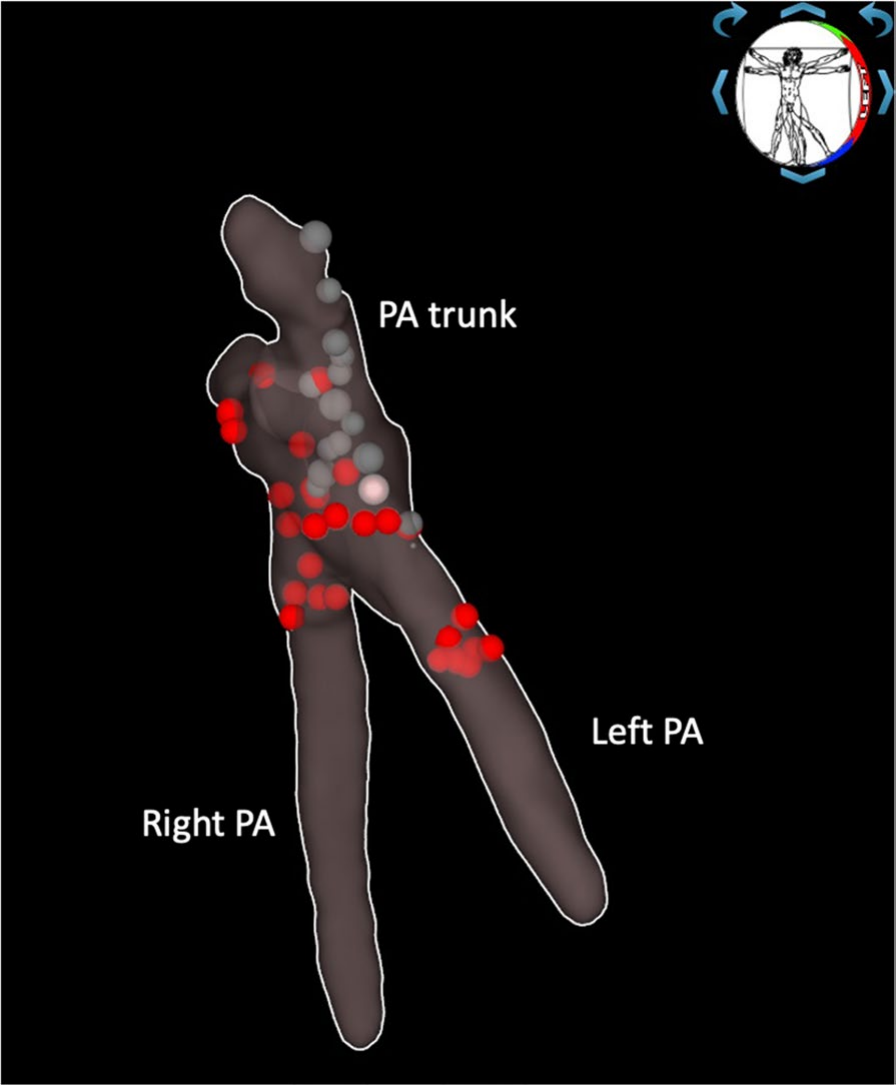
Three-dimensional electroanatomic map of the pulmonary artery with stimulation points where phrenic nerve was captured (gray points) and RF ablation points (red points)

Recurrent and phrenic nerves damage during PADN was prevented by PA stimulation mapping before each RF application as described previously [19]. Mapping and ablation were performed using a 3.5-mm tip open-irrigated ablation catheter (Celsius THERMOCOOL, Biosense Webster, CA, USA) under the guidance of the Biotok Unity electrophysiology and electroanatomic navigation system (Biotok, Tomsk, Russia); detailed technical aspects of the procedure were described elsewhere [19, 20]. RF ablation (40 W; application duration 30 s; irrigation 30 ml/min; RF power generator Biotok or ATAKR ® II RF, Medtronic, MN, USA) was performed in the PA trunk and proximal parts of the right and left PAs with the intention of circumferential ablations (excluding the sites with phrenic and recurrent laryngeal nerve capture while pacing; Fig. 2). In group I (PH-PADN), the PADN procedure was performed after a 20-min waiting period following complete restoration of mPAP to the baseline value after PH modeling-1.

#### PH modeling with TXA2

The target mPAP during PH modeling was 40 mmHg. PH was induced with continuous infusion of TXA2 (10 mg/ml; Tocris, Bristol, UK) through the left external jugular vein. The dose titration of the synthetic TXA2 analogue (U46619) was individual and adjusted in every animal. The dosage was increased gradually every 5 min in accordance with a predefined protocol (0.025, 0.05, 0.075, 0.1, 0.12, 0.15, 0.175 μg*kg^−1^*min^−1^) until the target mPAP [16]. Reversible PH modeling-1 was conducted after baseline hemodynamic assessment and reversible PH modeling-2 was performed 20 min after the PADN procedure. The dosage of TXA2 needed for the target mPAP was assessed before and after PADN in each animal. In group III (PH-shamPADN), PH modeling was conducted twice: the time difference between PH modeling-1 and 2 was 60 min to reproduce the time required for PADN in groups I and II.

#### Autopsy study and histological and immunohistochemical examination

After the experimental procedures, all animals were euthanized with an intracardiac injection of a lethal dose of potassium chloride. The heart and lungs were excised *en bloc* for gross anatomy assessment and histological examination of the PA trunk, left, and right PAs, and lungs at ablation sites. PA and its branches were transversely sampled into rings with a 5-mm step, and the samples were fixed in 10% buffered formalin, microscopy items were prepared routinely and stained with hematoxylin and eosin. Mallory trichrome staining (Biovitrum, Russia) was used to assess adventitia and perivascular adipose tissue in the areas of PA ablation. Immunohistochemical labeling was performed with antibodies for S100 protein (Dako Cytomation, Denmark) to detect nerve fibers as previously described [21]. The Leica Application Suite V 4.5.0 image analyzer and Leica Scope (Leica, Germany) were used for morphometric analysis. At the sites of identified RF lesions, the pulmonary artery was cut into rings (width 1 cm) which included a non-damaged area around the lesions. Each detected lesion was classified into grades I, II or III according to a previously published description [22]. The percentage of RF-induced lesions was calculated according to the following description: the area of a PA lesion divided by the total area of a PA ring specimen *100%. The percentage of a lesion was calculated separately for each lesion grade.

### Statistical analysis

Hemodynamic parameters (heart tare [HR], mBP, mPAP, RAP, PCWP, CO, PVR, and SVR) were analyzed to determine changes from the baseline and after PADN, between the PH modeling-1 and PH modeling-2. The TXA2 dosage and time to the target mPAP were compared during PH modeling-1 and 2 and after PADN. RF-induced PA lesions were presented as the percentage of the injured area. The association between the hemodynamic changes after PADN, in PH modeling-2 and the characteristics of RF-induced PA lesions were investigated. Data are presented as mean ± standard deviation (M±SD), absolute numbers and percentages. Mean values were compared using the Mann-Whitney U-test, Wilcoxon signed-rank test and Kruskal-Wallis ANOVA tests, as appropriate. Correlations were evaluated using the Spearman test. A statistically significant difference was determined as a two-tailed *P*<0.05. Statistical analysis of the data was carried out using Statistica for Windows, version 10.0 (StatSoft, USA).

## Results

The mean bodyweight of the animals was 36.2 ± 4.5 kg (age about 3 months). Baseline hemodynamic parameters are presented in Table 1. In two groups of animals where RF ablation was performed, the average number of ablation points was 23 ± 10.

**Table 1:** Baseline hemodynamic characteristics of the three groups (Kruskal–Wallis ANOVA, mean ± SD)

Parameters	All pigs, n = 17	Group I (PH-PADN), n = 9	Group II (non PH-PADN), n = 4	Group III (PH-shamPADN) n = 4	*p* value
HR, beat/min	97.5 ± 15.4	96.9 ± 11.0	97.8 ± 29.2	98.8 ± 9.1	0.86
SBP, mmHg	94.0 ± 11.5	93.1 ± 10.3	90.3 ± 12.6	99.8 ± 13.7	0.47
DBP, mmHg	54.0 ± 7.7	55.9 ± 5.8	50.5 ± 3.1	53.3 ± 13.7	0.21
mBP, mm Hg	66.8 ± 9.2	67.1 ± 8.5	64.3 ± 6.4	68.8 ± 14.3	0.80
SPAP, mm Hg	16.8 ± 3.9	17.2 ± 4.5	15.3 ± 3.3	17.3 ± 2.6	0.52
DPAP, mm Hg	12.0 ± 3.5	12.4 ± 3.8	10.3 ± 1.7	12.8 ± 4.3	0.58
mPAP, mm Hg	13.5 ± 3.7	13.9 ± 4.0	11.8 ± 2.5	14.5 ± 4.0	0.54
RAP, mm Hg	3.5 ± 2.2	3.9 ± 2.0	2.3 ± 1.5	4.0 ± 3.2	0.36
PCWP, mm Hg	4.4 ± 1.8	4.4 ± 2.1	3.8 ± 1.3	4.8 ± 1.7	0.74
CO, l/min	4.5 ± 1.5	4.7 ± 1.8	5.1 ± 1.3	3.4 ± 0.7	0.21
PVR, dyn/s/sm^*−5*^	186.6 ± 94.4	181.7 ± 85.4	139.7 ± 73.8	244.8 ± 122.8	0.37
SVR, dyn/s/sm^−5^	1252.7 ± 419.2	1213.0 ± 455.6	1018.0 ± 240.2	1576.5 ± 328.9	0.19

PH—pulmonary hypertension; PADN—pulmonary artery denervation; HR—heart rate; SBP—systolic blood pressure; DBP—diastolic blood pressure; mBP—mean arterial pressure; SPAP—systolic pulmonary arterial pressure; DPAP—diastolic pulmonary arterial pressure; mPAP—mean pulmonary artery pressure; RAP—right atrial pressure; PCWP—pulmonary capillary wedge pressure; CO—cardiac output; PVR—pulmonary vascular resistance; SVR—systemic vascular resistance

### Pulmonary artery denervation in group I (PH-PADN)

Hemodynamic parameters at baseline, and during PH induction-1 and PH-induction-2 are presented in Additional file 1: Table S1. TXA2 infusion was characterized by a dose-dependent target mPAP elevation due to significant PVR increase and accompanied with CO reduction. After reversible PH modeling-1 and subsequent pulmonary hemodynamics normalization, PADN was performed. The mean number of RF applications in the PA was trunk was 28.7 ± 16.5, in the right PA 8.6 ± 6.0, and in the left PA 8.2 ± 4.8.

The full study protocol with hemodynamic measurements was completed in 5 out of 9 animals. In three cases, pulmonary embolism (PE) was encountered: in animals #1 and 4 at the completion of the study protocol; in animal #6, PE was diagnosed and confirmed angiographically immediately after PADN, PH modeling-2 was not performed. In pigs #1 and 4 with PE, PH modeling-2 was characterized by lower doses of TXA2 and hemodynamic instability with profound blood pressure loss (Additional file 1: Table S1). In pig #8, TXA2 infusion was stopped during PH modeling-2 due to ventricular fibrillation and the data for PH modeling-2 in pig #8 as well as for animals with PE were removed from the analyses. No structural cardiac abnormality or PE was found in pig #8 on the autopsy study. Therefore, hemodynamic analysis after PADN in group I was performed on data obtained from 6 pigs, and hemodynamic data on PH modeling-2 was analyzed on 5 animals. Stable reversible PH modeling-2 was reproducible in all 5 animals and after TXA2 infusion termination, mPAP returned to its initial values within 15–20 min in all analyzed cases. The mean dose of TXA2 for the second PH induction was comparable to that used for the first induction.

### Pulmonary artery denervation in group II (nonPH-PADN)

The mean number of RF applications was 21.3 ± 0.5 and the number of RF applications in PA trunk (8.3 ± 0.9), left (6.5 ± 0.9) and right PAs (6.5 ± 0.9) was comparable to group I (*p* = 0.4; *p* = 0.2; *p* = 0.6; *p* = 0.4, respectively; Table 2). There was no PE in group II, and data from all 4 animals were included in analyses. No pulmonary nor systemic hemodynamic changes were noted after PADN in this normotensive group.

**Table 2:** Hemodynamic data after PADN during the normotensive period without the phase of PH induction in groups I (PH-PADN) and II (non-PH-PADN), (mean ± SD)

Parameters	Group I (PH-PADN) n = 6	Group II (nonPH-PADN) n = 4	*p* value*	Difference between baseline and after PADN		*P* value†
				Group (PH-PADN), n = 6	Group II (nonPH-PADN), n = 4	
Weight, kg	35.7 ± 2.6	39.5 ± 3.1	0.06	–	–	–
Ablation number	28.7 ± 16.5	21.3 ± 0.5	0.4	–	–	–
PA trunk ablation number	12.0 ± 6.1	8.3 ± 0.9	0.2	–	–	–
Left PA, ablation number	8.2 ± 6.0	6.5 ± 0.6	0.6	–	–	–
Right PA, ablation number	8.6 ± 4.8	6.5 ± 0.6	0.4	–	–	–
HR, beats/min	104.7 ± 17.2	95.0 ± 13.7	0.35	10.3 ± 20.5	−2.7 ± 15.6	0.46
SBP, mmHg	96.3 ± 7.7	83.5 ± 8.3	0.038	0.5 ± 16.3	−6.8 ± 8.8	0.26
DBP, mmHg	62.8 ± 11.1	42.0 ± 5.0	0.066	6.2 ± 13.9	−8.5 ± 6.9	0.06
mBP, mm Hg	74.0 ± 9.5	56.0 ± 4.3	0.066	6.0 ± 15.9	−8.3 ± 7.5	0.1
SPAP, mm Hg	18.7 ± 4.8	19.0 ± 8.8	0.61	-0.0 ± 3.0	3.8 ± 10.4	0.91
DPAP, mm Hg	11.8 ± 4.6	14.8 ± 7.6	0.47	−1.7 ± 3.9	4.5 ± 7.8	0.26
mPAP, mm Hg	14.2 ± 4.4	16.0 ± 8.1	0.91	−0.8 ± 3.4	4.3 ± 8.6	0.47
mRAP, mm Hg	3.5 ± 3.0	5.0 ± 4.2	0.47	−0.3 ± 3.7	2.8 ± 4.5	0.26
PCWP, mm Hg	4.0 ± 1.7	5.0 ± 3.4	0.91	−0.0 ± 2.3	1.3 ± 3.6	0.76
CO, l/min	4,6 ± 1,9	5.5 ± 1.7	0.61	−0.2 ± 1.1	0.4 ± 1,8	0.61
PVR, dyn/s/sm^*−5*^	214.8 ± 138.2	177.5 ± 102.8	0.76	14.1 ± 61.5	37.8 ± 125.0	1
SVR, dyn/s/sm^−5^	1474.6 ± 755.9	779.2 ± 141.1	0.17	318.9 ± 399.7	−238.8 ± 244.7	0.038

PH—pulmonary hypertension; PADN—pulmonary artery denervation; HR—heart rate; SBP—systolic blood pressure; DBP—diastolic blood pressure; mBP—mean arterial pressure; SPAP –systolic pulmonary arterial pressure; DPAP—diastolic pulmonary arterial pressure; mPAP—mean pulmonary artery pressure; RAP- right atrial pressure; PCWP—pulmonary capillary wedge pressure; CO—cardiac output; PVR—pulmonary vascular resistance; SVR—systemic vascular resistance

^*^ Mann–Whitney U two-sided exact test; † Wilcoxon signed-rank test

### Pulmonary hypertension modeling in group III (PH-shamPADN)

The target mPAP was achieved in all animals in group III (n = 4) and no signs of PE were detected. PH-modeling-2 was characterized by the same TXA2 doses and infusion time as the first PH modeling. The hemodynamic parameters between PH modeling-1 and -2 had no difference (Table 3). Therefore, there was no statistically detectable delayed effect of TXA2 on PVR or SVR during PH modeling-2.

**Table 3:** Hemodynamic data and the TXA2 dosage on PH modeling-2 in group I (PH-PADN) and group III (PH-shamPADN) (mean ± SD)

Parameters	Group I (PH-PADN), n = 6	Group III (PH-shamPADN), n = 4	*p* value*	Difference between PH 1 and PH 2 modeling		*P* value^†^
				Group I (PH-PADN), n = 6	Group III (PH-shamPADN), n = 4	
Weight, kg	35.7 ± 2.6	39.2 ± 1.9	0.038			
TXA2 dose, μg/kg for PH 1	20.1 ± 7.1	24.5 ± 5.7	0.35	3.25 ± 1.9	3.26 ± 8.3	0.5
TXA2 dose, μg/kg for PH 2	18.9 ± 6.7	21.2 ± 8.2	0.9			
Time to target mPAP-1, min	20.0 ± 11.0	18.8 ± 6.3	0.76	4 ± 2.2	2.5 ± 10.4	0.73
Time to target mPAP-2, min	18.3 ± 8.8	16.3 ± 9.5	0.61			
HR, beat/min	117.6 ± 20.6	115.8 ± 14.9	0.9	2.2 ± 10.6	−5.5 ± 16.5	0.5
SBP, mmHg	94.8 ± 13.8	104.0 ± 36.5	0.5	10.0 ± 10.9	−1.0 ± 37.7	0.5
DBP, mmHg	66.8 ± 12.5	62.3 ± 24.3	0.9	6.0 ± 6.5	−0.8 ± 27.0	0.7
mBP, mm Hg	76.2 ± 11.5	76.0 ± 27.9	0.9	7.2 ± 8.1	−0.5 ± 30.0	0.7
SPAP, mm Hg	44.6 ± 5.0	42.8 ± 1.0	0.28	1.2 ± 4.3	−1.5 ± 3.1	0.28
DPAP, mm Hg	34.4 ± 2.9	38.0 ± 0.8	0.06	−1.2 ± 1.8	−1.0 ± 1.4	0.9
mPAP, mm Hg	38.2 ± 2.5	39.8 ± 0.5	0.5	0.0 ± 1.2	−1.5 ± 1.7	0.2
mRAP, mm Hg	7.2 ± 1.9	5.3 ± 2.2	0.28	−2.2 ± 1.6	1.0 ± 0.8	0.015
PCWP, mm Hg	5.6 ± 0.6	6.8 ± 10	0.11	−1.2 ± 0.4	0.3 ± 1.3	0.06
CO, l/min	3.0 ± 1.5	2.4 ± 0.7	1	0.8 ± 1.5	0.3 ± 1.4	0.9
PVR, dyn/s/sm^−5^	740.9 ± 421.2	1228.9 ± 524.3	0.4	−171.6 ± 874.5	107.7 ± 798.2	0.9
SVR, dyn/s/sm^−5^	2091.8 ± 800.8	2373.7 ± 692.8	0.7	10.0 ± 623.1	101.0 ± 549.0	0.9

PH—pulmonary hypertension; PADN—pulmonary artery denervation; HR—heart rate; SBP—systolic blood pressure; DBP—diastolic blood pressure; mBP—mean arterial pressure; SPAP –systolic pulmonary arterial pressure; DPAP—diastolic pulmonary arterial pressure; mPAP—mean pulmonary artery pressure; RAP—right atrial pressure; PCWP—pulmonary capillary wedge pressure; CO—cardiac output; PVR—pulmonary vascular resistance; SVR—systemic vascular resistance

*Mann–Whitney U two-sided exact test

^†^Wilcoxon signed-rank test

### Pulmonary artery denervation and hemodynamics during normotensive periods in groups I (PH-PADN) and II (nonPH-PADN)

There was no statistically significant change in pulmonary pressures, and CO immediately after PADN in the entire cohort of pigs with PADN (n = 10); as well as in separate analyses within groups I and II (Table 2).

**Fig. 3:**
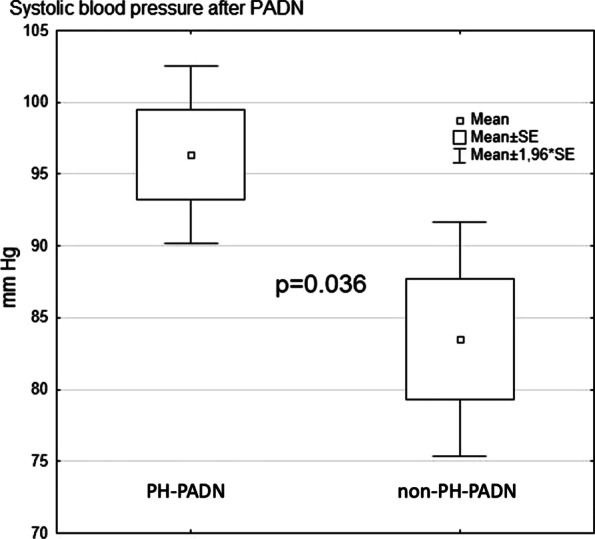
Systolic BP decrease in group II (nonPH-PADN) after PADN

At the same time, there was a significant SBP (*p* = 0.036) drop and a trend toward lower DBP and mBP (*p* = 0.06; *p* = 0.06, respectively) in group II (nonPH-PADN) compared to group I (PH-PADN) immediately after PADN (Fig. 3). The differences between DBP and SVR at the baseline and following PADN were significant in group II (nonPH-PADN) (Table 2).

No meaningful correlations were found between the number of RF applications in PA and pressure values after PADN in the all PADN pigs (mBP: ρ = 0.031; SPAP: ρ = 0.376; DPAP: ρ = 0.22; mPAP: ρ = 0.36; PCWP: ρ = -0.23; RAP: ρ = 0.28; CO: ρ = -0.32; SVR: ρ = 0.10; PVR: ρ = 0.42; *p* > 0.05 for all parameters). However, in group I (PH-PADN), positive correlations were noted between the number of RF ablations and HR, SPAP and RAP immediately after PADN (ρ = 0.82, *p* = 0.02; ρ = 0.67, *p* = 0.02; ρ = 0.62, *p* = 0.049, respectively), as well as a PVR difference between baseline and after PADN (ρ = 0.64, *p* = 0.02, Additional file 1: Figure S1). No significant correlations between the number of RF applications and hemodynamic parameters were noted in group II (nonPH-PADN).

### Pulmonary arterial hypertension modeling-2 in group I (PH-PADN) and group III (PH-shamPADN)

**Fig. 4:**
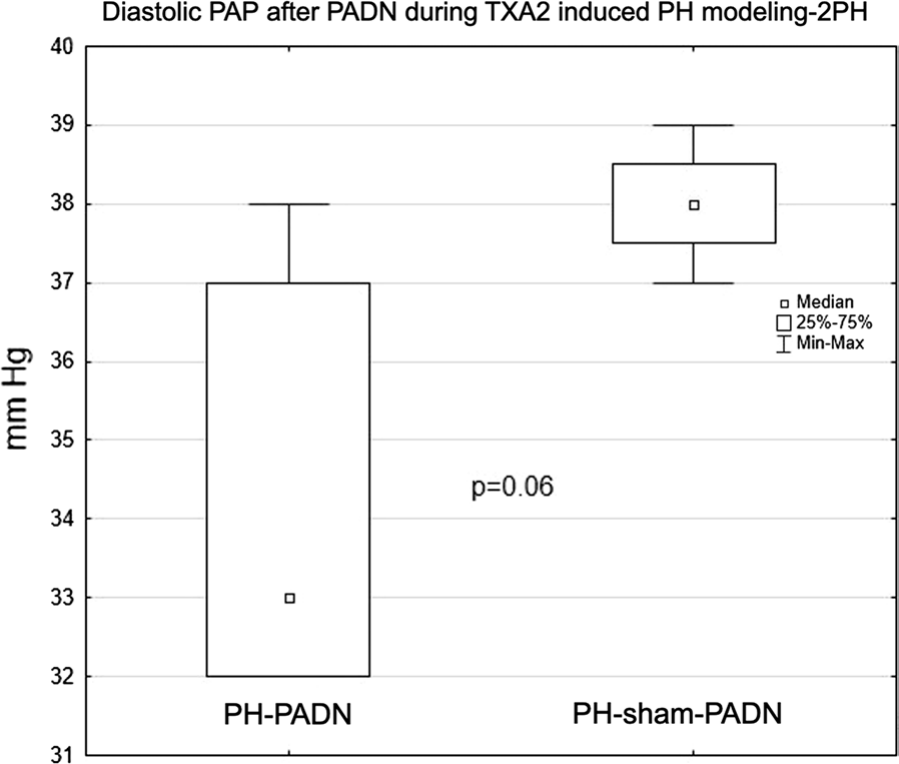
Diastolic PAP during TXA2-induced PH modeling-2 in group I (PH-PADN) and group III (PH-shamPADN)

The TXA2 dose and the time to the target mPAP in PH modeling-1 and 2 did not differ between groups I (PH-PADN) and III (PH-shamPADN): 20.1 ± 7.1 vs 24.5 ± 5.7 μg/kg, *P* = 0.35; 18.3 ± 8.8 Vs 16.3 ± 9.5 min, *P* = 0.61, respectively. Within each group, there was no change in a consumed TXA2 dose for the second PH modeling (Table 3). At PH modeling-1, the PCWP was higher in group III (PH-shamPADN) than in group I (PH-PADN): 7.0 ± 1.8 vs 4.3 ± 0.8 mm Hg, *P* = 0.02. Diastolic PAP tended to decrease after PADN during PH modeling-2 (*p* = 0.06) when compared with the PH-shamPADN group (Fig. 4). In group III (PH-shamPADN), PH modeling-2 was characterized by a higher RAP difference between the PH modeling-1 and -2 when compared with group I (PH-PADN), (1.0 ± 0.8 Vs -2.2 ± 1.6 mm Hg, *P* = 0.015).

A positive correlation was observed between the number of RF applications and the HR during PH modeling-2 (ρ = 0.9; *p* = 0.037).

### Artery wall lesion severity and hemodynamic changes after pulmonary artery denervation

The percentage of the area with grade I lesions was higher in group I (PH-PADN) when compared with group II (nonPH-PADN): 50 ± 13.5 vs 20.2 ± 15.0%; *P* = 0.038 (Table 4). No significant correlation was detected between the lesion percentages and hemodynamic changes after PADN in group II (nonPH-PADN) (HR: ρ = 0.94; SBP: ρ = 0.60; DBP: ρ = 0.8; mBP: ρ = 0.6; SPAP: ρ = −0.4; DPAP: ρ = 0; mPAP: ρ = −0.21; PCWP: ρ = −0.40; RAP: ρ = −0.31; CO: ρ = 0.40; PVR: ρ = −0.20; SVR: ρ = −0.20, respectively; *p* > 0.05 for all parameters).

**Table 4:** Summary of the lesions percentage in group I and group II. (mean ± SD)

Lesion grade percentage (%)	Group I (PH-PADN) (n = 6)	Group II (nonPH-PADN) (n = 4)	*P* value*
All lesions	94.3 ± 49.8	110 ± 43.7	0.61
Grade I	20 ± 15	50 ± 13.5	0.038
Grade II	25.8 ± 8.6	30 ± 19	0.61
Grade III	45.8 ± 31.5	40 ± 8.7	0.9
Grade II + III	74 ± 35.7	60 ± 34.8	0.47

*Mann–Whitney U two-sided exact test

**Fig. 5:**
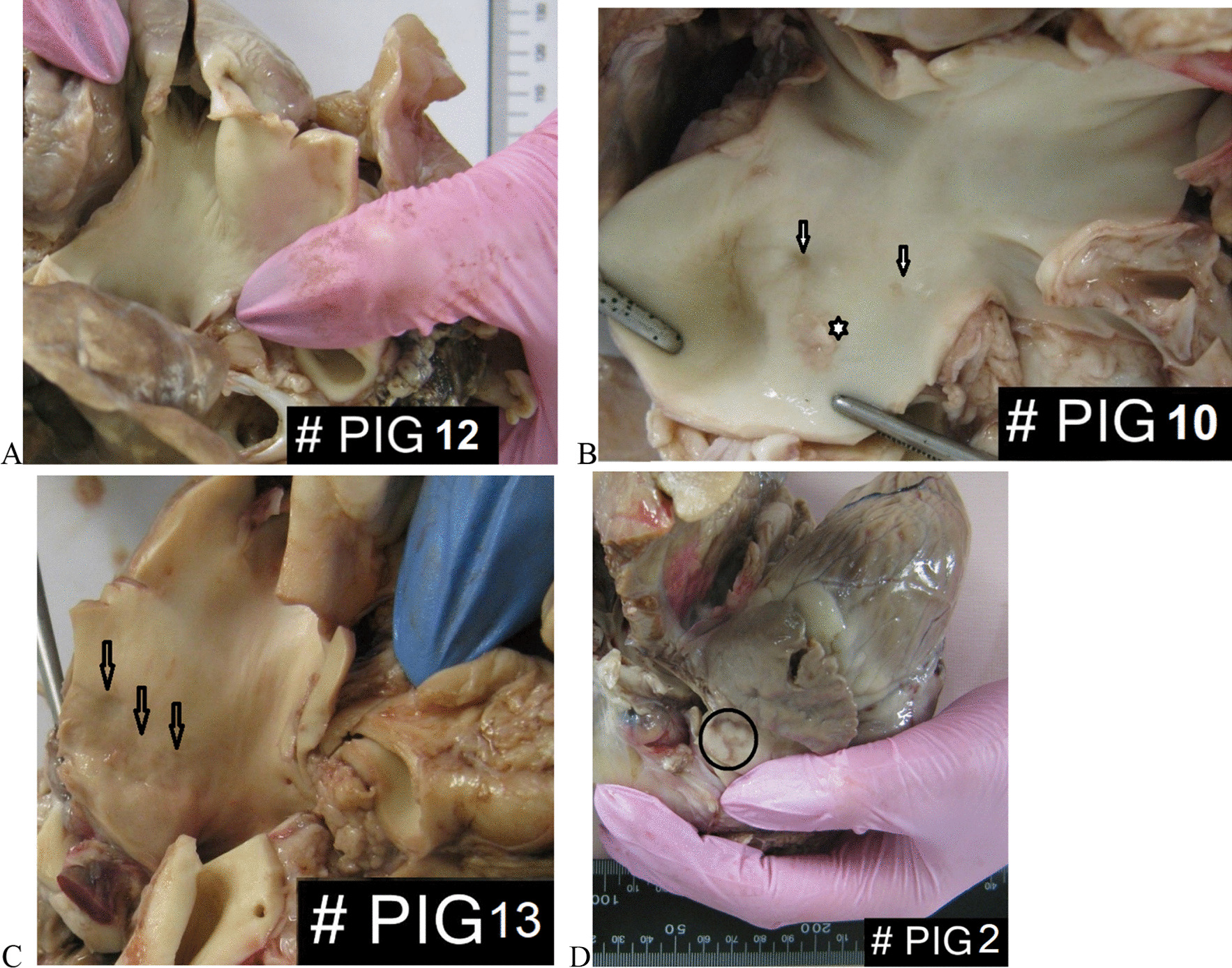
Macroscopic evaluation of RF-induced PA lesions: **a**. No lesions observed in the PA trunk, left and right PAs; **b** and **c**. Brown spots (arrows) depict PA wall hemorrhages and rough defects (4 mm-3 mm-2 mm in size); the asterisk corresponds to superficial dissection with intima detachment and media disorganization; **d** Macroscopic view of a RF-induced lesion at the PA trunk (circle) corresponds to coagulation necrosis of adventitia, perivascular fat and nerve fibers with intact underlying intima and media on further histological examination

**Fig. 6:**
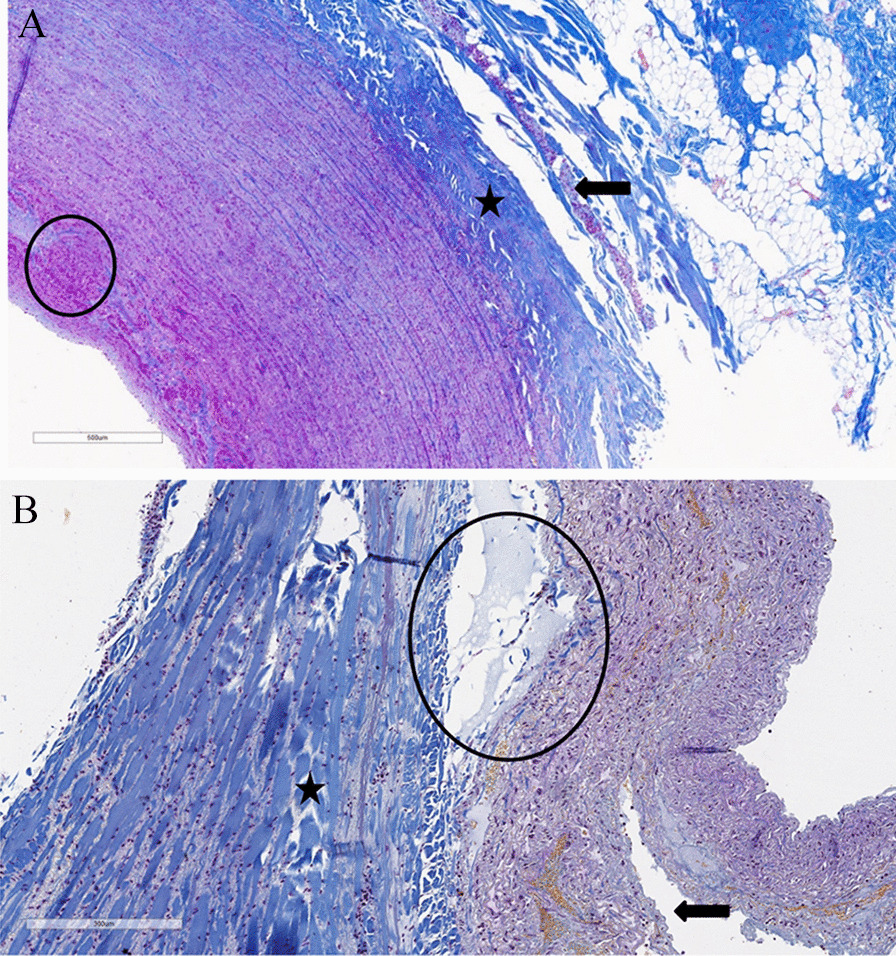
Histological study of percutaneous RF-induced PA wall lesion: **a** Coagulation necrosis (asterisk) of adventitia with underlying unaltered nerve fiber (arrows), edema in 1/3 media (circle), × 50**; b** RF-induced dissection (arrow) with transmural PA wall lesion without underlying nerve fibers (asterisk), × 50

In the animals after PADN (PH-PADN and nonPH-PADN groups, n = 10), RF-induced PA lesions were inhomogeneous (Fig. 5), here fore, a semi-quantitative grading system for each detected RF lesion was applied (Additional file 1: Table S2) [22]. The percentages of the lesion grades are presented in Additional file 1: Table S3. S100 expression was preserved in the majority of the PA specimens with RF ablation, even in cases with the most severe adventitia lesions—grade III (Fig. 6). A strong positive correlation was evident between the type III lesion percentage in the PA and the number of RF applications (ρ = 0.74, *p* = 0.013). A positive correlation was observed between the mean total RF lesion area per PA specimen and the animal weight (ρ = 0.65, *p* = 0.04). There was a trend toward a negative correlation between SBP, DBP after PADN and the mean total RF lesion area per a PA specimen (ρ = −0.61, *p* = 0.059); and a positive correlation with the DBP difference between the baseline value and after PADN (ρ = 0.65, *p* = 0.039). No significant correlation was found between type III lesion percentage and hemodynamic changes after PADN. The presence of a higher percentage of grade II and III lesions in PA was associated with HR acceleration after PADN (ρ = 0.68, *p* = 0.03). A negative correlation was noted between grade I lesion percentage and diastolic BP (ρ = −0.77, *p* = 0.01) after PADN.

Groups I (PH-PADN) and II (nonPH-PADN) showed no difference in the total percentage of RF-induced lesions in the PA (*p* = 0.61) (Table 4). A negative correlation was noted between the percentage of all RF lesions and the DBP and mBP differences in PH modeling-2 (ρ = −0.90; *p* = 0.037, ρ = -0.90; *p* = 0.037, respectively). The percentage of grade III lesions in PA was not associated with hemodynamic changes after PADN, the TXA2 dose, or the time needed for PH modeling-2. A tendency toward a positive correlation between grade III percentage and SVR (ρ = 0.87, *p* = 0.053) was found in PH modeling-2, moreover, the positive correlation with SVR during PH modeling-2 reached a statistically significant value when the percentage of grade II and III lesions were analyzed together (ρ = −0.90; *p* = 0.037).

## Discussion

The results of our experimental study with reversible PH induction show that PADN does not lead to acute changes in mean PAP. The PADN procedure does not influence the TXA2 dosage and times to reach the target mean PAP during repeat PH induction. The more RF applications delivered to the PA wall, the higher number of deeper lesions with adventitial damage is found. The number of deep PA lesions is associated with HR acceleration immediately after PADN.

No substantive correlation has been observed between the number of RF applications in the PA and hemodynamic changes after PADN in the entire cohort of animals. However, the number of RF applications in group I (PH-PADN) has been associated with a significant HR, systolic PAP, RAP increase, and PVR elevation after PADN. The percentage of grade II and III lesions in the PA is associated with the degree of HR acceleration and the SVR elevation during PH modeling-2. These hemodynamic changes after PADN could be attributed to a sensitizing effect of TXA2 on the vasculature. HR elevation might be associated with the extent of perivascular nerve damage, as seen in cardiac arrhythmia ablation procedures [23]. Therefore, we suggest that HR acceleration after PADN might be a sign of extensive PA lesions and more effective denervation. In animals without PH modeling, PADN leads to a significant SBP drop associated with SVR decrease. A possible mechanism of systemic BP fluctuations during PADN could be attributed to the interaction between pulmonary arterial and carotid sinus baroreceptor reflexes unless general anesthesia and TXA2 could modify natural response [24, 25].

One of our key findings is that the number of RF applications positively correlates with the percentage of grade III PA lesions. This suggests that a small number of discrete PA ablations cannot be reaching the majority of perivascular neural fibers, and more excessive ablation is required for denervation. Additionally, despite the high number of severe PA wall lesions, no acute nerve fiber necrosis is observed at the sites of catheter unipolar RF ablation. We used even higher RF power (40 W) than other authors [17, 18, 26]. Thus, our previous report on experimental data in swine has demonstrated that 40 W RF ablation induces deep lesions penetrating to PA nerve fibers [27]. The current study results show preserved nerves despite rough PA wall damage at ablation sites. Rothman et al. applied 2–8 RF ablations (> 55 °C; impedance reduction > 10%; > 60 s) to the PA trunk and its bifurcation and found a reduction in S100 expression at the sites of ablation that corresponded to the mPAP and PVR decrease in TXA2-induced acute PH in swine. We have applied a greater number of RF applications with higher energy, and no correlation of the number and lesion grade have been found associated with changes in PH induction. Possible causes of insufficient PADN effect and nerve damage include the absence of tight constant contact between the ablation catheter tip and the PA wall during RF application, insufficient duration of applications (30 versus 60 and 120 s in the other studies), and the absence of continuous circumferential damage in our experiment. However, the presence of RF-induced PA lesions suggests that catheter position and orientation are adequate in the majority of applications. The application of specially designed catheters can be more appropriated for denervation. Thus, Chen et al. established a loop-shaped ablation catheter with 10 electrodes and, using RF energy (temperature 50° C, energy ≤ 10 W, impedance < 140 Ω, time 20 s.) was able to completely abolish the PH induced by PA balloon occlusion in dogs [28].

Although our results suggest that in the acute phase no physical destruction of perivascular fibers could be found, we speculate that in chronic experiments, delayed nerve damage might be expected. Thus, Rothman et al. demonstrated hemodynamic improvement in patients with pulmonary arterial hypertension (PAH) at 4 months after ultrasound PADN, whereas no significant changes in hemodynamic parameters were noted immediately after the denervation procedure [8]. Zhou et al. have recorded the effect of nerve fiber degeneration 3 months after RF-PADN in dogs [26], however, Garcia Lunar has shown that transcutaneous RFA is not accompanied by a significant PA nerve destruction or transmural PA wall lesions and they observed intact PA walls near the RF application site. By contrast, surgical PADN with bipolar radiofrequency clamps (with tissue impedance monitoring for transmurality control) has led to nerve fiber damage but has not been associated with a PAP decrease in chronic pulmonary venous hypertension (PVH) either. No changes in histology or function (MRI) of the heart and pulmonary vessels are registered 3 months after surgical PADN with bipolar RF clamps in swine [17]. The reason for the absence of PAP decrease after PADN in the Lunar study could be attributed to the PVH model used. Nevertheless, the PAP decrease has been demonstrated after PADN in the majority of clinical studies which included patients with PAH of different origins and even in pulmonary venous hypertension patients with heart failure [5].

PA stimulation mapping of PA zones associated with sympathetic autonomic nerve response has been proposed for more targeted PA denervation [19]. However, the benefits of targeted vs non-targeted PA denervation have not been proven so far [20, 29]. In the present study, excessive PA ablation has been performed with the avoidance of sites close to the phrenic and laryngeal nerves. The number of RF ablations delivered to the PA has been significantly associated with an increase in systolic PAP immediately after PADN in swine with prior PH modeling-1. We suggest that TXA2 infusion and PA wall damage by RF ablation may sensitize the pulmonary vasculature to vasoconstriction, thereby leading to the systolic PAP and PVR increase directly after PADN. Duggan et al. have demonstrated that PA in swine is prone to vasoconstriction reactions and displays PA neurogenic contraction irrespective of vessel caliber on electrical stimulation [30]. Nevertheless, the trend to the diastolic PAP decrease in PH modeling-2 suggests the possible effect of PADN on acute PH inducibility. However, the latter assumption remains speculative.

The mechanism of the PAP decrease after PADN in patients with PAH is still unclear as severely remodeled PAs exhibit extremely low vasodilatation properties and nerve fibers density gradually decreases towards the periphery [31]. In this setting, acute PH modeling without severe histological changes in the PAs could be relevant for the evaluation of the acute PADN effect on PA vasodilatation as the probable mechanism of the mean PAP decrease. Previous reports demonstrated that PADN may potentially affect PH induction by TxA2, a potent vasoconstrictor and pro-inflammatory agent (Rothman et al. [18]). It is assumed that PADN affects the afferent neural regulation of pulmonary vasculature tonus and abolishes PA vasospasm, and, as a consequence, leads to a decrease in PA pressure in an acute period after denervation. Acute pulmonary artery pressure decrease was registered immediately after PADN in the majority of experimental and clinical studies in PH. The lack of vasoconstriction despite repeat TXA2 infusion might be the only possible mechanism of the presumptive positive effect of PADN in this model. Another PH model that has been used in other studies is chronic hypoxic PH, which is characterized by typical for PH pulmonary arteries remodeling characterized by media hypertrophy, fibrosis, and PA lumen narrowing with low vasodilative properties. Therefore, in the hypoxic PH model possibly weaker response to PADN had been expected. Whereas, TxA2 infusion leads to an acute and reversible mean PAP elevation without typical for chronic PH arterial remodeling.

Another issue is RF-induced PA wall injury with subsequent PA remodeling as well as the concomitant damage of parasympathetic nerve fibers that lie together with sympathetic ones [21, 32] and mediate the PA vasodilatation effect [33]. Various types of micro-injuries including PA wall dissection, coagulation of media and adventitia, parietal thrombosis, and hemorrhages in different layers at the sites of RF applications and adjacent areas have been revealed on pathomorphological examination in swine [22, 27]. In chronic PVH, no significant PA wall changes have been noted 3 months after transcutaneous RF PADN [17]. Moreover, it is still unclear whether PADN influences PA remodeling on its length in swine. Unfortunately, the data on PA remodeling after PADN in rats with monocrotaline PH model [9] could not be transferred directly to humans or even to swine as they exhibit completely different PA innervation patterns and neurogenic responses to the adrenergic stimulation [30].

Pulmonary embolism could be another potential problem associated with PADN. Three animals from the PH-PADN group have shown acute PE with pulmonary hemodynamic deterioration. In all cases, PADN has been performed after complete normalization of hemodynamics after the termination of the first TXA2 infusion, unless platelets could remain activated. Our study implemented additional two groups: the group of PADN without TXA2 infusion and the group with double TXA2 PH induction with a sham procedure. This allows assessing the possible influence of PADN on PA thrombus generation. No PE has been revealed on the autopsy in these groups; nevertheless, we are not able to exclude a thrombogenic effect of the PADN with TXA2 infusion, which, along with vasoconstriction, activates platelets [34]. The mean number of ablation points in studies by Rothman A. et al. and Zhou et al. is less than in our study but even limited RF ablation number might lead to endothelial damage and thrombus formation predisposition. The anticoagulation protocol was not described in the study by Rothman A. et al. We used intravenous heparin with an empirical target activated clotting time ≥ 300 s, but even this level of anticoagulation might not be enough when extensive PA endothelial damage is created. Rothman A. et al. depicted postmortem in situ thrombi at the ablation sites in PA without providing the number of animals with thrombi. Another important factor is the difference in TXA2 dosing (from 1.5 to 10.5 mcg/kg per hour in our study and from 17 to 37 mcg/kg per hour in the study by Rothman et al.); in our study, much lesser doses were used with the same target mPAP. One may note that TXA2 formulation and manufacturers were different in our study and study by Rothman A et al., suggesting different bioactivity of the agent. We suggest that the high rate of pulmonary embolism in our series might have complex reasoning, including extended PA ablation and greater TXA2 activity.

Along with previous experimental and clinical studies on PADN, the present report demonstrates the complexity of determining PADN procedure endpoints and prediction of possible subsequent effects. The establishment of the intraoperative criteria for PADN effectiveness and appropriate subject selection requires a meticulous analysis of acute and postponed hemodynamic changes. Transmurality of RF-induced PA damage is not associated with PAP changes in the acute PH modeling study or chronic pulmonary venous hypertension in swine [17]. Effective and less traumatic energy sources with accurate focusing on the targeted area may therefore be desirable for targeting PA nerves. Ultrasonic or laser energy application for PADN could potentially lead to significant nerve damage with lesser endothelial trauma and only a minimal effect on the medial layer. [35].

### Limitations

The acute design of the present study does not allow assessment of the morphological and hemodynamic changes in a delayed period after PADN.

The small samples in this study only provide statistical power to detect strong correlations, and possible moderate correlations may be underestimated.

The full study protocol with hemodynamic measurements was completed in 5 out of 9 animals allocated to group I. One may expect some effect of the reduction in sample size on the reproducibility of our findings, but this reduction was expected when planning the experiment, as stated in methods, and the resulting number of animals in three groups was comparable.

We cannot fully exclude TxA2 toxic effects and delayed implication on PH induction during the second challenge. Although there was no statistically meaningful difference in TxA2 dose during the second challenge when compared to the first infusion, the numerical difference has a trend toward a shorter induction time. Moreover, the high rate of pulmonary embolism found in group I may be related to the combination of pro-thrombotic effects of TXA2 infusion itself and PA endothelium damage due to extensive RF ablation.

A conventional RF ablation catheter may not be fully adequate for PA denervation procedures and lead to intima damage in a high proportion of lesions; however, histologically high grades of PA damage have been found in the majority of RF applications.

Acute PH modeling with synthetic TXA2 may not be fully relevant for interventions on pulmonary arteries as the delayed vascular wall and platelet sensibilization could influence PADN effects. The TXA2 model of PH cannot reflect all forms of PH where PADN may still be effective in PAP reduction.

## Conclusions

In the acute experiment in swine, transcatheter PADN does not affect PH inducibility using TXA2. The number of RF applications delivered to the PA wall has a positive correlation with the percentage of high-grade wall lesions affecting the adventitia and perivascular adipose tissue. Significant PA adventitia damage is associated with HR acceleration after PADN. The potential effects of PADN on PH inducibility in a chronic experiment require further investigation.

## Supplementary Information

**Additional file 1**. Supplementary materials (Table S1, Table S2, Table S3, Figure S1).

## Data Availability

The datasets used and/or analyses during the current study are available from the corresponding authors on reasonable request.

## Abbreviations

CO: Cardiac output
DBP: Diastolic blood pressure
DPAP: Diastolic pulmonary artery pressure
HR: Heart rate
mBP: Mean blood pressure
mPAP: Mean pulmonary artery pressure
PA: Pulmonary artery
PADN: Pulmonary artery denervation
PAH: Pulmonary arterial hypertension
PAP: Pulmonary artery pressure
PCWP: Pulmonary capillary wedge pressure
PH: Pulmonary hypertension
PVR: Pulmonary vascular resistance
RAP: Right atrial pressure
RHC: Right heart catheterization
RF: Radiofrequency
SBP: Systolic blood pressure
SPAP: Systolic pulmonary artery pressure
SVR: Systemic vascular resistance
TXA2: Thromboxane A2
