# Supplementary material for: Transcatheter radiofrequency pulmonary artery denervation in swine: the evaluation of lesion degree, hemodynamics and pulmonary hypertension inducibility
Source: BMC Pulm Med. 2021 Dec 18;21:418. doi: 10.1186/s12890-021-01786-y (PMC8684280; doi:10.1186/s12890-021-01786-y)
Supplement: Supplementary file 1 — Additional file 1. Supplementary materials (Table S1, Table S2, Table S3, Figure S1). [file 12890_2021_1786_MOESM1_ESM.docx]

**Transcatheter Radiofrequency Pulmonary Artery Denervation in Swine: the Evaluation of Lesion Degree, Hemodynamics and Pulmonary Hypertension Inducibility**

Natalia S. Goncharova, MD, PhD, Heber Ivan Condori Leandro, MD, Aleksandr D. Vakhrushev, MD, Elena G. Koshevaya, MD, Yury A. Skorik, PhD, Lubov B. Mitrofanova MD, PhD, Lada A. Murashova, VMD, Lev E. Korobchenko, Elizaveta M. Andreeva, Dmitry S. Lebedev, MD, PhD, Olga M. Moiseeva, MD, PhD, Evgeny N. Mikhaylov, MD, PhD

# Almazov National Medical Research Centre, Saint-Petersburg, Russian Federation

**ADDITIONAL FILE 1. Supplementary materials (Table S1, Table S2, Table S3, Figure S1).**

**Table S1.** Hemodynamic parameters at baseline, after PADN, during PH modeling-1 and PH modeling-2.

|  | **Baseline/PADN/PH1/PH2 TXA2 dose (mcg*kgг^-1^)**  **and time to target mPAP (min)** | | | | | | **HR,**  ***beats/min.*** | **mBP,**  ***mm Hg*** | **mPAP, *mm Hg*** | **mRAP,**  ***mm Hg*** | **PCWP,**  ***mm Hg*** | **SVO_2_,**  ***%*** | **CO,**  ***l/min*** | **PVR,**  ***dynes/s/sm^-5^*** | **SVR,**  ***dynes/s/sm^-5^*** |
| --- | --- | --- | --- | --- | --- | --- | --- | --- | --- | --- | --- | --- | --- | --- | --- |
| **Pig №1**** | baseline | | | | | | 86 | 60 | 12 | 5 | 5 | 65 | 2.2 | 251.2 | 1974.0 |
|  | PADN, ablation number=13 | | | | | | 79 | 60 | 13 | 6 | 6 | 62 | 2.05 | 272.7 | 2104.3 |
|  |  | Time | | TXA2 dose | | |  |  |  |  |  |  |  |  |  |
|  | PH1 | 20 | | 43.5 | | | 98 | 69 | 45 | 12 | 7 | 55 | 1.7 | 1753.6 | 2630.3 |
|  | PH2 | 5 | | 14.5 | | | 108 | 36 | 20 | 4 | 4 | 51 | 1.13 | 804 | 1608 |
| **Pig №2** | baseline | | | | | | 95 | 69 | 14 | 4 | 5 | 78 | 3.6 | 198.1 | 1430.7 |
|  | PADN, ablation number=14 | | | | | | 92 | 75 | 12 | 3 | 5 | 75 | 3.19 | 175.1 | 1800.8 |
|  | PH1 | 35 | | 27.9 | | | 94 | 71 | 37 | 5 | 3 | 69 | 2.6 | 1054.5 | 2047 |
|  | PH2 | 30 | | 23.9 | | | 90 | 70 | 35 | 5 | 5 | 66 | 2.4 | 1020.5 | 2211 |
| **Pig №3** | baseline | | | | | | 84 | 56 | 11 | 5 | 4 | 82 | 5.5 | 100.8 | 734.4 |
|  | PADN, ablation number=14 | | | | | | 91 | 77 | 11 | 3 | 6 | 86 | 7.1 | 56 | 828.8 |
|  | PH1 | 25 | | 25.5 | | | 109 | 102 | 40 | 5 | 5 | 81 | 5.3 | 532 | 1474.4 |
|  | PH2 | 25 | | 25.5 | | | 108 | 90 | 40 | 8 | 6 | 83 | 5.65 | 462.4 | 1115 |
| **Pig №4**** | Baseline | | | | | | 112 | 67 | 7 | 0 | 2 | 84 | 4.3 | 92.2 | 1235.8 |
|  | PADN, ablation number=17 | | | | | | 98 | 68 | 16 | 5 | 4 | 88 | 5.78 | 165.9 | 871.5 |
|  | PH1 | 25 | | 21 | | | 109 | 85 | 40 | 9 | 7 | 87 | 5.3 | 494.5 | 1139 |
|  | PH2 | 25 | | 21 | | | 94 | 65 | 40 | 8 | 6 | 79 | 3.5 | 823 | 1379.8 |
| **Pig №5** | baseline |  | |  | | | 90 | 73 | 18 | 5 | 4 | 87 | 7.3 | 152.5 | 740.5 |
|  | PADN, ablation number=15 | | | | | | 102 | 78 | 13 | 2 | 2 | 83 | 5.61 | 156.6 | 1082.3 |
|  | PH1 | 25 | | 21.3 | | | 128 | 78 | 39 | 6 | 5 | 83 | 5.6 | 484.2 | 1025.4 |
|  | PH2 | 20 | | 17.75 | | | 115 | 60 | 40 | 7 | 6 | 60 | 2.38 | 1139 | 1776 |
| **Pig № 6*** | baseline | | | | | | 108 | 69 | 16 | 7 | 9 | 87 | 6.4 | 87.3 | 773.5 |
|  | PADN, ablation number=18 | | | | | | 108 | 59 | 26 | 9 | 12 | 87 | 6.4 | 174.6 | 623.7 |
|  | PH1 | 20 | | 14.5 | | | 129 | 91 | 42 | 11 | 13 | 89 | 7.6 | 306.1 | 844.5 |
|  | PH2 was not conducted due to PE during PADN | | | | | | | | | | | | | | |
| **Pig № 7** | baseline | | | | | | 111 | 55 | 11 | 3 | 2 | 54 | 2.3 | 311.5 | 1799 |
|  | PADN, ablation number=35 | | | | | | 105 | 78 | 14 | 9 | 5 | 38 | 2.01 | 356.9 | 2737 |
|  | PH1 | 20 | | | 22.98 | | 150 | 79 | 35 | 7 | 4 | 41 | 1.38 | 1791.8 | 4161 |
|  | PH2 | 15 | | | 19.15 | | 142 | 81 | 36 | 10 | 5 | 39 | 1.71 | 1447.2 | 3314 |
| **Pig № 8** | baseline | | | | | | 98 | 76 | 16 | 4 | 5 | 82 | 5.7 | 153.4 | 1004 |
|  | PADN, ablation number=40 | | | | | | 100 | 55 | 12 | 0 | 4 | 77 | 3.2 | 133 | 733 |
|  | PH1 | 5 | | | 9.725 | | 142 | 76 | 43 | 5 | 2 | 67 | 6 | 954 | 1736.9 |
|  | PH2 wasn’t conducted due to ventricular fibrillation | | | | | | | | | | | | | | |
| **Pig № 9** | baseline | | | | | | 88 | 70 | 20 | 2 | 4 | 73 | 4,4 | 288 | 1224 |
|  | PADN, ablation number=45 | | | | | | 138 | 80 | 23 | 4 | 4 | 69 | 3.69 | 410.9 | 1665 |
|  | PH1 | 10 | | | 13.2 | | 118 | 87 | 40 | 2 | 5 | 70 | 3.9 | 700.2 | 1700 |
|  | PH2 | 5 | | | 8.4 | | 133 | 81 | 40 | 6 | 6 | 60 | 2.7 | 990.6 | 2156 |
| **Pig № 10** | Baseline | | | | | | 75 | 59 | 15 | 4 | 5 | 67 | 3.2 | 249.8 | 1373.9 |
|  | PADN, ablation number=21 | | | | | | 85 | 56 | 14 | 1 | 3 | 79 | 5.12 | 171.7 | 858.7 |
| **Pig № 11** | Baseline | | | | | | 75 | 59 | 11 | 3 | 4 | 81 | 5.25 | 106.57 | 852.6 |
|  | PADN, ablation number=21 | | | | | | 85 | 58 | 28 | 11 | 10 | 77 | 4.5 | 317 | 828.6 |
| **Pig № 12** | Baseline | | | | | | 106 | 72 | 9 | 1 | 2 | 82 | 6.02 | 93 | 942.8 |
|  | PADN, ablation number=21 | | | | | | 114 | 60 | 11 | 4 | 4 | 86 | 7.87 | 71 | 568 |
| **Pig № 13** | Baseline | | | | | | 105 | 67 | 12 | 1 | 4 | 78 | 5.85 | 109 | 902.4 |
|  | PADN, ablation number=22 | | | | | | 96 | 50 | 11 | 4 | 3 | 74 | 4.27 | 149.7 | 860.7 |
| **Pig № 14** | Baseline | | | | | | 87 | 69 | 18 | 5 | 7 | 73 | 4.33 | 203.13 | 1181.8 |
|  | PH1 | | 25 | | | 22.9 | 115 | 77 | 40 | 4 | 5 | 70 | 3.20 | 874.66 | 1824.3 |
|  | PH2 | | 10 | | | 15.5 | 126 | 87 | 40 | 4 | 6 | 66 | 2.71 | 1003.5 | 2449.7 |
| **Pig №15** | baseline | | | | | | 109 | 88 | 11 | 8 | 5 | 76 | 3.56 | 134.89 | 1798.5 |
|  | PH1 | | 20 | | | 22.1 | 114 | 75 | 43 | 8 | 6 | 77 | 3.46 | 855.91 | 1549.8 |
|  | PH2 | | 15 | | | 18.4 | 95 | 40 | 39 | 6 | 6 | 35 | 1.31 | 2009.3 | 2070.2 |
| **Pig №16** | baseline | | | | | | 101 | 64 | 18 | 1 | 4 | 69 | 2,66 | 420.3 | 1891.5 |
|  | PH1 | | 10 | | | 17.8 | 98 | 70 | 41 | 4 | 8 | 49 | 1.47 | 1802.8 | 3605.5 |
|  | PH2 | | 10 | | | 17.8 | 115 | 106 | 40 | 3 | 8 | 67 | 2.49 | 1024.8 | 3298.6 |
| **Pig №17** | Baseline | | | | | | 98 | 54 | 11 | 2 | 3 | 65 | 2.90 | 220.6 | 1434.3 |
|  | PH1 | | 20 | | | 28.3 | 114 | 80 | 41 | 9 | 9 | 56 | 2.69 | 951.4 | 2110.8 |
|  | PH2 | | 30 | | | 33.4 | 127 | 71 | 40 | 8 | 7 | 78 | 3.00 | 8779.8 | 1676.1 |

**Footnote:** *- thrombotic occlusion of the main left PA confirmed with angiopulmonography immediately after PADN. PH modeling-2 was not conducted. ** - pulmonary embolism revealed on autopsy study; PH 1 – pulmonary hypertension induction using cоntinious TXA2 infusion before PADN; PH2 - pulmonary hypertension induction using cоntinious TXA2 infusion 20 minutes after PADN; PADN – pulmonary artery denervation; HR - heart rate; mBP - mean arterial pressure; mPAP - mean pulmonary artery pressure; mRAP- mean right atrial pressure; PCWP – pulmonary capillary wedge pressure; PVR - pulmonary vascular resistance; CO - cardiac output; SVR - systemic vascular resistance.

**Table S2.** Semiquantitative assessment of ablation lesions [22].

| **Grade** | **Histology findings** | **Comment** |
| --- | --- | --- |
| Grade III | Hemorrhages at any depth of the media, in the adventitia and adipose tissue (with a diameter more than 800 µm).  Dissections of the artery wall with a depth more than 50% of the media thickness.  Coagulation necrosis of perivascular adventitia and adipose tissue with/without damage to nerve fibers. | Total transmural lesion that involves all possible neural structures within the artery wall and/or in adventitia. |
| Grade II | Focal hemorrhages at any depth of the media, in the adventitia and adipose tissue (with a diameter of 200-800 µm).  Focal necrosis of the intima and the inner third of the media (depth up to 10% of the thickness of the media layer).  Dissections of the artery wall with a depth of up to 50% of the media thickness. | Non-transmural lesion that involves part of neural structures within the artery wall or in adventitia. |
| Grade I | Focal edema of the inner third of the media and extending to a depth of up to 10% of the PA wall thickness.  Small-focal hemorrhages at any depth of the media and in the adventitia (up to 200 µm in diameter). | PA wall lesion without any evidence of nerve damage. |

μm = micrometre

**Table S3.** Summary of RF induced lesion characteristics in the swine pulmonary artery revealed by histological and immunohistochemical examination.

|  | **PA Trunk** | | | | **Right PA** | | | | **Left PA** | | | |
| --- | --- | --- | --- | --- | --- | --- | --- | --- | --- | --- | --- | --- |
|  | **#Ablation number** | **# Lesion grade** | | | **#Ablation number** | **# Lesion grade** | | | **#Ablation number** | **# Lesion grade** | | |
|  |  | **Lesion % in specimen** | | |  | **Lesion % in specimen** | | |  | **Lesion % in specimen** | | |
|  |  | **I** | **II** | **III** |  | **I** | **II** | **III** |  | **I** | **II** | **III** |
| **Pig №1*** | 6 | 1 | 10 | 20 | 4 | 5 | 15 | 0 | 3 | 5 | 10 | 0 |
| **Pig №2** | 7 | 5 | 10 | 30 | 4 | 5 | 5 | 0 | 2 | 1 | 5 | 0 |
| **Pig №3** | 6 | 5 | 10 | 15 | 5 | 5 | 10 | 0 | 3 | 0 | 5 | 0 |
| **Pig №4*** | 5 | 0 | 5 | 25 | 4 | 0 | 5 | 0 | 8 | 0 | 10 | 0 |
| **Pig №5** | 7 | 5 | 10 | 15 | 4 | 5 | 10 | 0 | 4 | 5 | 10 | 0 |
| **Pig №6*** | 6 | 1 | 5 | 10 | 5 | 5 | 5 | 0 | 7 | 5 | 20 | 0 |
| **Pig №7** | 15 | 15 | 10 | 15 | 12 | 0 | 5 | 15 | 10 | 20 | 25 | 45 |
| **Pig №8**** | 17 | 30 | 15 | 20 | 15 | 5 | 0 | 10 | 14 | 0 | 0 | 75 |
| **Pig №9** | 20 | 5 | 0 | 0 | 18 | 0 | 10 | 5 | 18 | 15 | 10 | 45 |
| **Pig №10** | 9 | 15 | 0 | 5 | 6 | 0 | 0 | 25 | 6 | 15 | 0 | 0 |
| **Pig №11** | 8 | 35 | 20 | 0 | 6 | 5 | 0 | 0 | 7 | 15 | 5 | 0 |
| **Pig №12** | 7 | 15 | 15 | 5 | 7 | 15 | 10 | 25 | 7 | 30 | 20 | 15 |
| **Pig №13** | 9 | 20 | 10 | 30 | 7 | 25 | 15 | 10 | 6 | 10 | 20 | 5 |

Footnote:*-pigs with pulmonary embolism were excluded from the analyses of PADN effect on hemodynamics; ** - the pig with ventricular fibrillation was excluded from the analyses of PADN effect on PH modeling-2; PA –pulmonary artery; RF – radiofrequency.

**ABC**


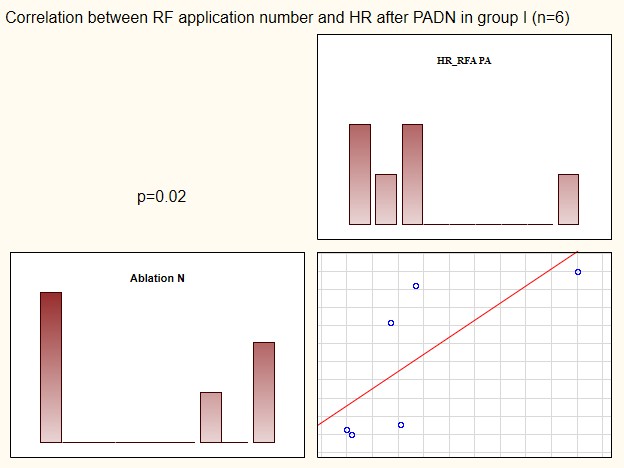

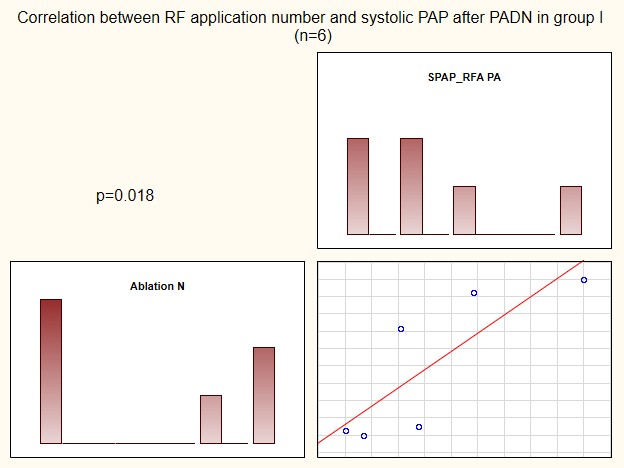

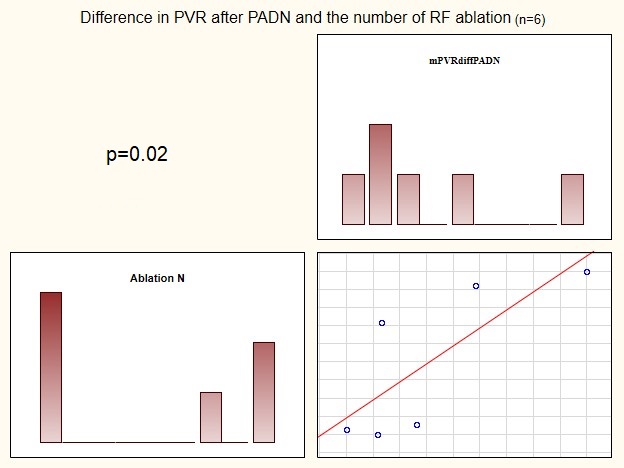


# Figure S1. Correlations between the RF application number and A. HR, B. Systolic PAP and C. PVR elevation after PADN in group I (PH-PADN, n=6).
